# Nonunion of a scapular spine fracture: Case report and management with open reduction, internal fixation, and bone graft

**DOI:** 10.4103/0973-6042.42202

**Published:** 2008

**Authors:** Mohammed As-Sultany, Amol Tambe, David I. Clark

**Affiliations:** Derbyshire Royal Infirmary, London Road, Derby, UK; 1 Wrightington Upper Limb Unit, Wrightington, UK; 2 Derbyshire Royal Infirmary, Derby, UK

**Keywords:** Fracture, internal fixation, nonunion, open reduction, scapular spine

## Abstract

Fractures of the scapular spine are relatively uncommon. We report a case of a 39 year old male who developed an atrophic non-union scapular spine fracture entering the spino-glenoid notch. We describe our experience with this rare fracture pattern and identify the need for early internal fixation in the young, active and working population.

## INTRODUCTION

Scapular fractures are relatively uncommon and generally represent 0.5–1% of all fractures.[1] Of these, fractures of the body and neck are the most common and account for more than two-thirds of the cases, with intra-articular fractures of the glenoid cavity (rim and fossa) making up approximately 10%.[2] Fractures of the acromial and coracoid processes account for 9% and 7%, respectively, while those of the scapular spine only represent about 6%.[3,4]

We describe a case of an uncommon fracture of the scapular spine, with the fracture extending into the spino-glenoid notch. The minimal displacement of this fracture at initial presentation led us to treat this injury conservatively. However, it went on to develop atrophic nonunion and required exploration, internal fixation, and autogenous bone grafting to alleviate the patient's pain and disability. From our experience with this rare fracture pattern and the ensuing complication, we feel that a case can be made for early internal fixation of these fractures in the young, active, and working population.

## CASE REPORT

**Figure 1:**
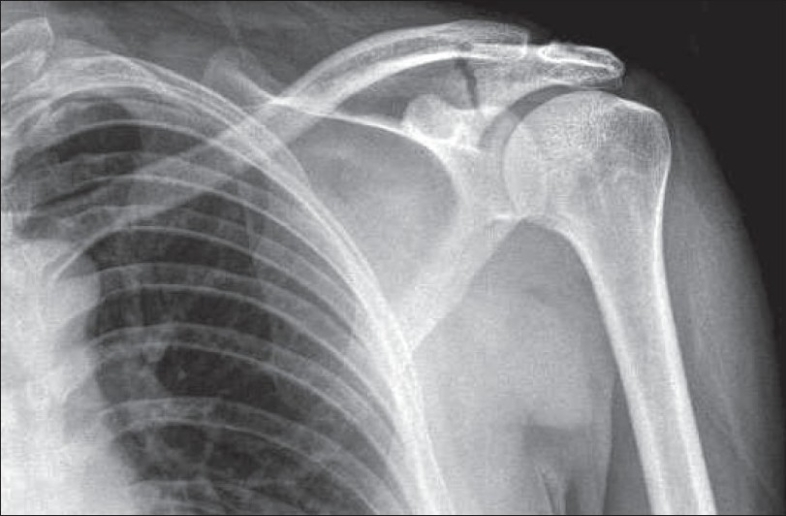
Initial radiograph of the scapular spine fracture

A 39-year-old male patient presented to us in June 2006 with isolated left shoulder pain after he had fallen directly onto the shoulder whilst taking part in paintballing. On clinical examination, he had soft tissue swelling, with tenderness and bruising over the lateral aspect of the scapular spine. Passive and active abduction and forward flexion of the left shoulder were uncomfortable beyond 90°, with well-maintained internal and external rotation of the arm. Although the motor power of the rotator cuff muscles appeared grossly normal, there was evidence of mild inhibition of the rotator cuff function due to pain when muscle power was tested against resistance. There was no neurovascular compromise to the left upper limb. Radiograph of the left shoulder revealed a minimally displaced scapular spine fracture at the base of the acromion and entering the spino-glenoid notch [Figure 1].

**Figure 2:**
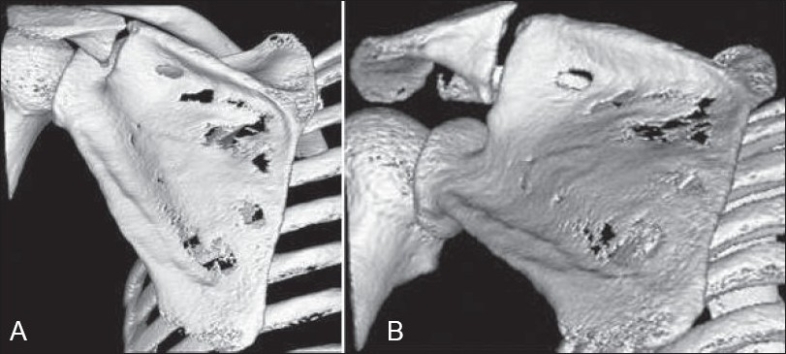
Three-dimensional reconstruction CT of the scapular spine fracture: (A) PA view and (B) oblique view

The patient consented to conservative treatment, which involved resting the arm in a polysling and using analgesic medication for pain relief. He was regularly followed-up in the fracture clinic and began a range of gradual movements after 4 weeks. However, he continued to have symptoms of aching and mobility at the fracture site, which started to affect his quality of life and his ability to carry out routine daily work and leisure activities. Follow-up radiographs failed to show any evidence of healing even after 6 months. A CT scan carried out at this stage clearly demonstrated nonunion at the fracture site [Figure 2].

In view of his ongoing problems the patient was offered open reduction and internal fixation with bone grafting of the scapular spine fracture. After appropriate discussion, the patient gave informed consent for the procedure.

## SURGICAL TECHNIQUE AND POSTOPERATIVE MANAGEMENT

The patient was placed in a lateral decubitus position and the bony prominences were padded adequately. Standard preparation of the left arm, shoulder, scapula, and left iliac crest was carried out. The incision was made one finger-breadth above and parallel to the spine of scapula, extending from 6 cm medial to the palpable fracture site to the posterolateral corner of the acromion. The deltoid-trapezius fascia was opened along the line of the scapular spine using sharp dissection. The trapezius insertion over the nonunion fracture site and the superior lip of the scapular spine were lifted off using subperiosteal dissection. Care was taken to ensure that there was minimal disruption of the deltoid muscle origin along the inferior lip of the scapular spine. Minimal dissection of the supraspinatus muscle belly was required to expose the nonunion, which was mobile and atrophic in nature. All atrophic tissue was removed with a curette down to fresh bleeding bone and a 2-mm drill bit was used to further open the sclerotic bone edges. Care was taken at all times to avoid transgressing into the spino-glenoid notch.

**Figure 3:**
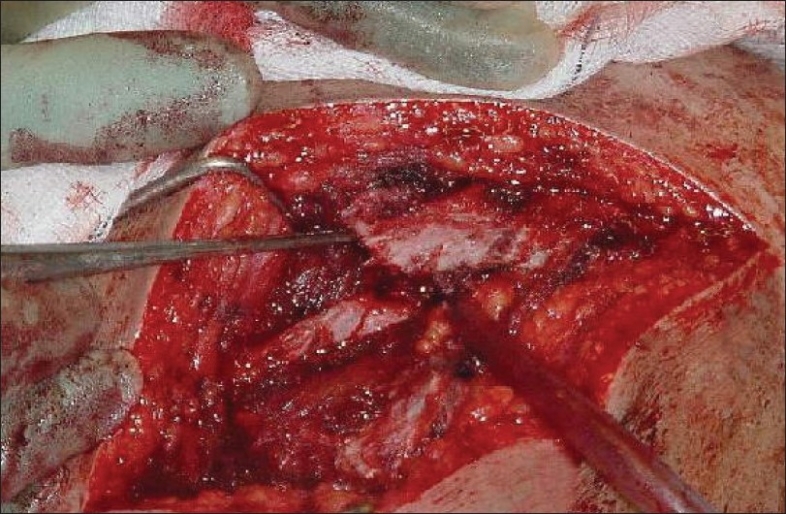
Perioperative identification of the scapular spine fracture

**Figure 4:**
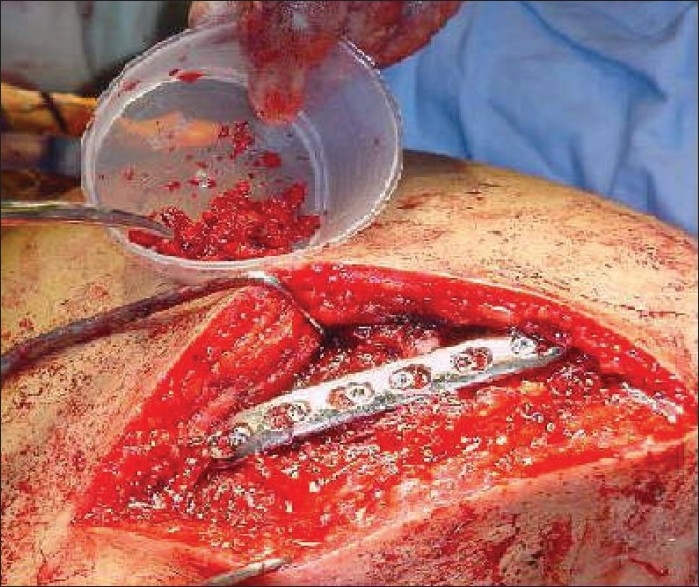
Fixation of the 6-hole small-fragment LCDCP with bone grafting

Meanwhile, cancellous bone graft was harvested from the left iliac crest using a bone trephine and a curette. A six-hole small fragment low-contact dynamic compression plate (LCDCP) was contoured to the spine of the scapula such that it sat slightly above the scapular spine. It was fixed to the ridge of the scapular spine in this position with three cortical screws on either side of the nonunion. The harvested bone graft was then packed into both sides of the fracture [Figures 3 and 4]. The deltoid and trapezius muscles were repaired with nonabsorbable sutures, using the thick insertional tissue to buttress the repair. Once the wound was dressed and the left arm placed in a polysling with a chest strap, the patient was woken up. Perioperative check radiographs showed satisfactory medial and lateral plate fixation across the fracture site.

**Figure 5:**
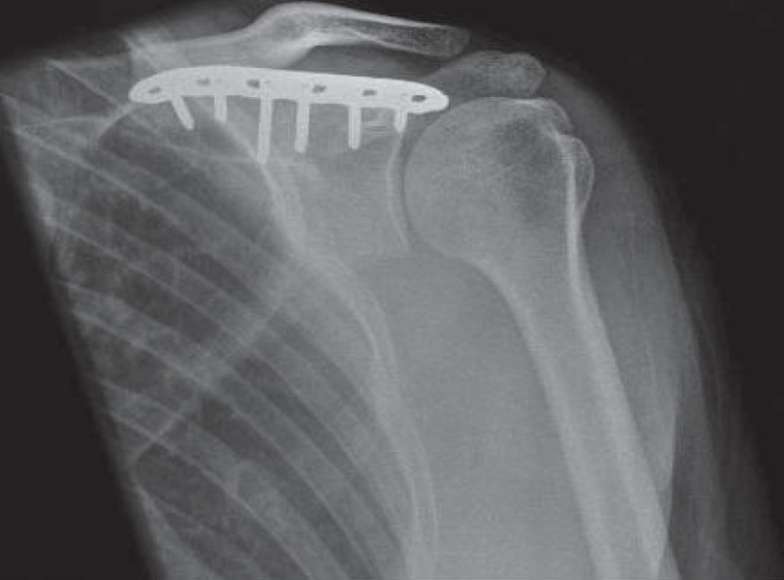
Complete union of the scapular spine fracture 3 months after surgery

There were no immediate postoperative complications and the patient was discharged a day later in his polysling, which was worn for 4–5 weeks to rest the shoulder. During this time he was instructed to commence hand, wrist, and elbow exercises, stabilizing his arm against the chest wall. After 2 weeks the patient began gentle pendulum exercises under the supervision of physiotherapists. Passive shoulder abduction up to 60° and gentle assisted rotations with the arm by the side were commenced after 3 weeks. Increasing range of active assisted movements was undertaken between 5 and 6 weeks, along with the use of pulleys and bands in a graded fashion. Since the radiograph taken during the sixth week demonstrated good plate position with satisfactory ongoing consolidation, abduction beyond 90° was allowed. Although after 8 weeks the patient had commenced independent daily activity with abduction below 90°, he was not allowed to carry out any manual or heavy activity before 3 months had elapsed. He was also strongly advised not to play any sports until there was unequivocal evidence of fracture consolidation. The 3-month postoperative radiograph demonstrated complete fracture healing, with satisfactory implant position [Figure 5]. More importantly, the patient's initial symptoms of mobility and pain at the fracture site had disappeared and he was able to return to work and perform light desk duties. He continued to make excellent progress and had full painless function in the shoulder girdle at his last review 5 months post surgery and subsequently returned to full work duties as a shopkeeper. A recent evaluation using the 12-item Oxford questionnaire (self-administered)[5] revealed a very satisfactory score of 14. He only had very mild and intermittent discomfort directly over the plate area when lying flat or sitting back against a hard-back chair. However it was not severe enough to warrant implant removal.

## DISCUSSION

Scapular spine fractures, especially those at the base of the acromion, are uncommon. Furthermore, the complex bony anatomy of the scapula sometimes makes accurate classification of fractures on a plain radiograph rather difficult, thus justifying the need to use CT scanning.[6] The use of three-dimensional reconstructed CT images in the current case provided essential and accurate preoperative information about the position and extent of the fracture, thus facilitating preoperative planning.

We are currently unaware of any major studies that have specifically focused upon scapular spine fractures, possibly because such fractures are frequently grouped with fractures of the scapular body or acromion. Those fractures that enter the spino-glenoid notch are clearly different from isolated acromial fractures and therefore should be identified and treated differently.[7]

Scapular spine fractures are generally a result of high-energy trauma and tend to be nondisplaced and treated nonoperatively.[8] In our patient, the injury happened as a result of direct force from the posterior or posterolateral direction as the patient landed on the posterior aspect of his shoulder. We decided to treat it conservatively according to the currently accepted practice. However, nonunion of the scapular spine at the base of the acromion caused persistent pain and significant limitation of function. We believe that the sagging of the lateral spine and acromion effectively produced narrowing of the supraspinatus outlet and secondary impingement of the rotator cuff. Continuous lateral traction due to the weight of the arm also produced fatigue and pain in his trapezius muscle.

The classification system proposed by Ogawa *et al.*[9] considered scapular spine fractures as an extension of acromion fractures. They emphasized that the majority of displaced symptomatic fractures distal to the acromial angle can be successfully treated with Kirschner wire and tension-band wiring. On the other hand, it is more appropriate to use plate fixation for more proximal and medially displaced fractures involving the spine. This was further supported by Robinson *et al.*,[10] who reported a case of a symptomatic non-union fracture of the lateral scapular spine, which was successfully treated with open reduction and internal fixation. Recently, Shindle *et al.*[11] described two cases of stress fracture involving the scapular spine in chronic rotator cuff tear arthropathy. They utilized plate fixation in the first case, whereas they used a reverse-geometry shoulder replacement for the advanced arthrosis in the second case. Similarly, intra-articular glenoid cavity and displaced scapular neck fractures are traditionally managed by open reduction and internal fixation as they have the potential to disrupt the mechanics of the gleno-humeral joint.[1,2,12,13] Operative management is also typically reserved for displaced acromial process fractures to prevent the occurrence of subacromial impingement and pseudoarthrosis.[14]

The current case, though uncommon, is not unique. It highlights the need to perhaps have a low threshold for operative fixation of such (undisplaced) fractures, especially in young, fit, and active patients.
